# Coexistence of lung cancer and immunoglobulin G4-related lung disease in a nodule: a case report

**DOI:** 10.1186/s13256-016-0898-3

**Published:** 2016-05-09

**Authors:** Hiroki Tashiro, Koichiro Takahashi, Tomomi Nakamura, Kazutoshi Komiya, Shinya Kimura, Naoko Sueoka-Aragane

**Affiliations:** Division of Hematology, Respiratory Medicine and Oncology, Department of Internal Medicine, Faculty of Medicine, Saga University, 5-1-1 Nabeshima, Saga, 849-8501 Japan

**Keywords:** IgG4-related disease, Lung cancer, Lung nodule

## Abstract

**Background:**

Immunoglobulin G4-related disease is characterized by infiltration of immunoglobulin G4-positive plasmacytes in various organs. The radiological findings of lung involvement of immunoglobulin G4-related disease include hilar and mediastinal lymphadenopathies, thickness of bronchovascular bundles, peribronchovascular consolidation, and lung nodules. Although a pathological approach is needed to diagnose immunoglobulin G4-related disease, it is ordinarily diagnosed by biopsy from one lesion even if there are multiple lesions. We reported a rare case of the coexistence of immunoglobulin G4-related disease and lung cancer in the same lung nodule.

**Case presentation:**

A 72-year-old Japanese man visited our hospital for evaluation of a nodular shadow in the middle lobe of his right lung that was seen on chest radiograph and computed tomography scan. An abdominal computed tomography scan showed a tumefactive lesion in his anterior sacral spine. Blood examinations revealed high serum immunoglobulin G4 concentration at 346 mg/dl, renal dysfunction, and anemia. He underwent right upper lobectomy and regional lymph node dissection. Pathologic findings of the lung nodule showed lepidic pattern adenocarcinoma with infiltration of immunoglobulin G4-positive plasma cells and obliterative phlebitis.

**Conclusions:**

To date, there have been only few reports on the coexistence of immunoglobulin G4-related disease and lung cancer; here, we report such a rare case. Histologic examination should be considered in cases of suspicious immunoglobulin G4-related disease appearing in a lung nodule.

## Background

IgG4-related disease is characterized by infiltration of IgG4-positive plasmacytes in various organs [1]. The clinical manifestations include autoimmune pancreatitis, Riedel’s thyroiditis, tubulointerstitial nephritis, Mikulicz’s disease, and retroperitoneal fibrosis; histologic findings show lymphoplasmacytic infiltration, obliterative phlebitis, and storiform fibrosis [2]. The patterns of lung involvement of IgG4-related disease include hilar and mediastinal lymphadenopathies, thickness of bronchovascular bundles, peribronchovascular consolidation, and lung nodules. Here, we report a rare case of lung cancer coexisting with IgG4-related disease in the same lung nodule. It is interesting that the cancer cells and IgG4-positive plasma cells coexisted in the same nodule, rather than being just a complication.

## Case presentation

**Fig. 1:**
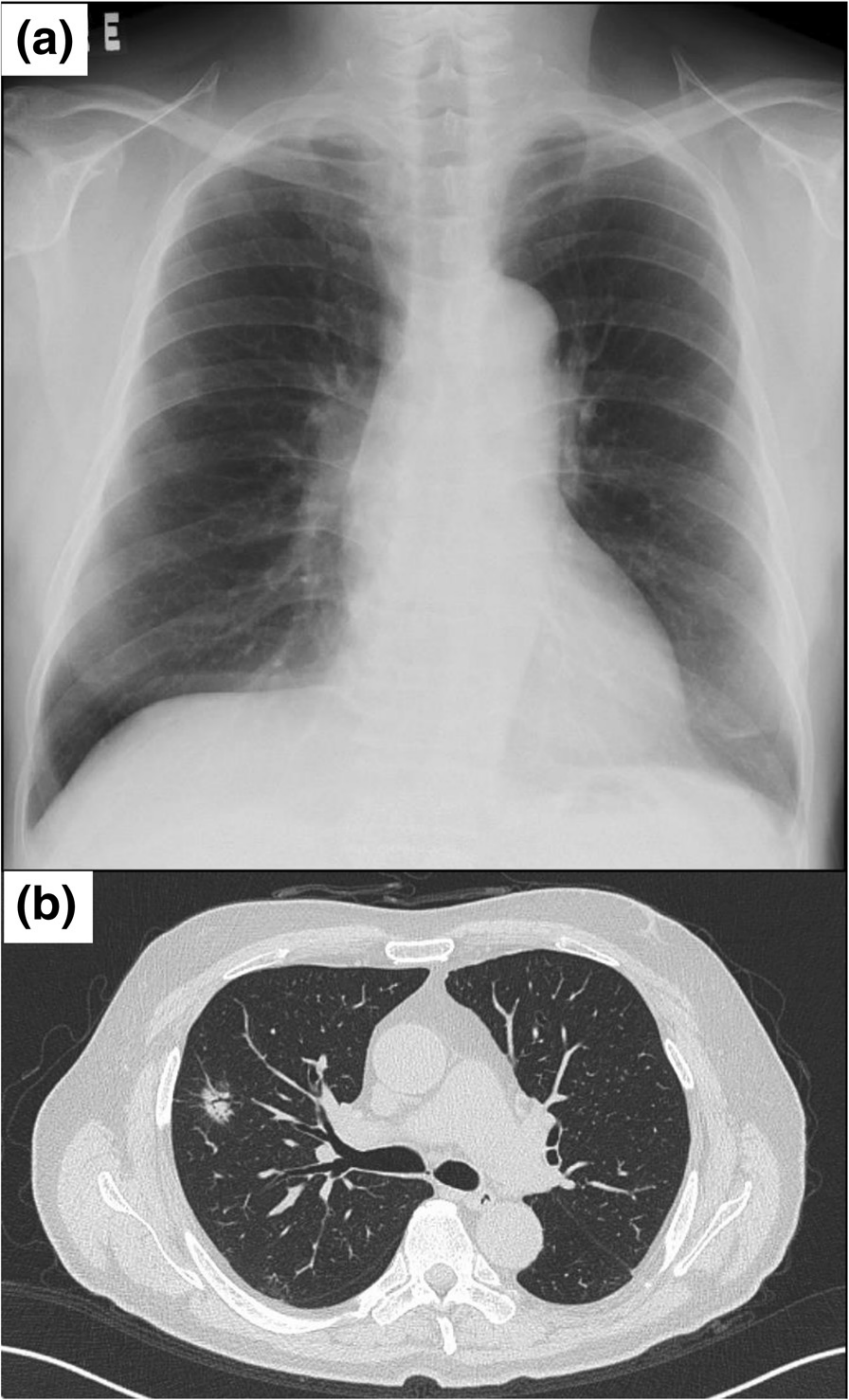
The findings of a chest radiograph and computed tomography. **a** Chest radiograph shows a nodular shadow in the middle lobe of his right lung. **b** Chest computed tomography revealed a 20-mm spiculated part-solid nodule with pleural indentation in segment 3 on the right

A 72-year-old Japanese man, who had no underlying disease, visited our hospital because of a nodular lung shadow that was detected during an annual health examination. He did not have any respiratory symptoms, such as cough, sputum production, and dyspnea. A chest radiograph showed a small nodular shadow in the middle lobe of his right lung (Fig. 1a). A chest computed tomography (CT) scan showed a 20-mm spiculated nodule with pleural indentation in the upper lobe of his right lung (Fig. 1b), but no hilar and mediastinal lymphadenopathies were seen. An abdominal CT scan showed a tumefactive lesion in his anterior sacral spine and left hydronephrosis. His laboratory findings on admission were 10.8 g/dl hemoglobin, 27.1 mg/dl blood urea nitrogen, 2.04 mg/dl creatinine and 0.53 mg/dl C-reactive protein. His IgG and IgG4 concentration was at 2407 mg/dl and 346 mg/dl (normal range, 4.8 to 105 mg/dl). Tumor markers, such as carcinoembryonic antigen (CEA) and cytokeratin fragment (CYFRA) 21–1, were within normal range.

**Fig. 2:**
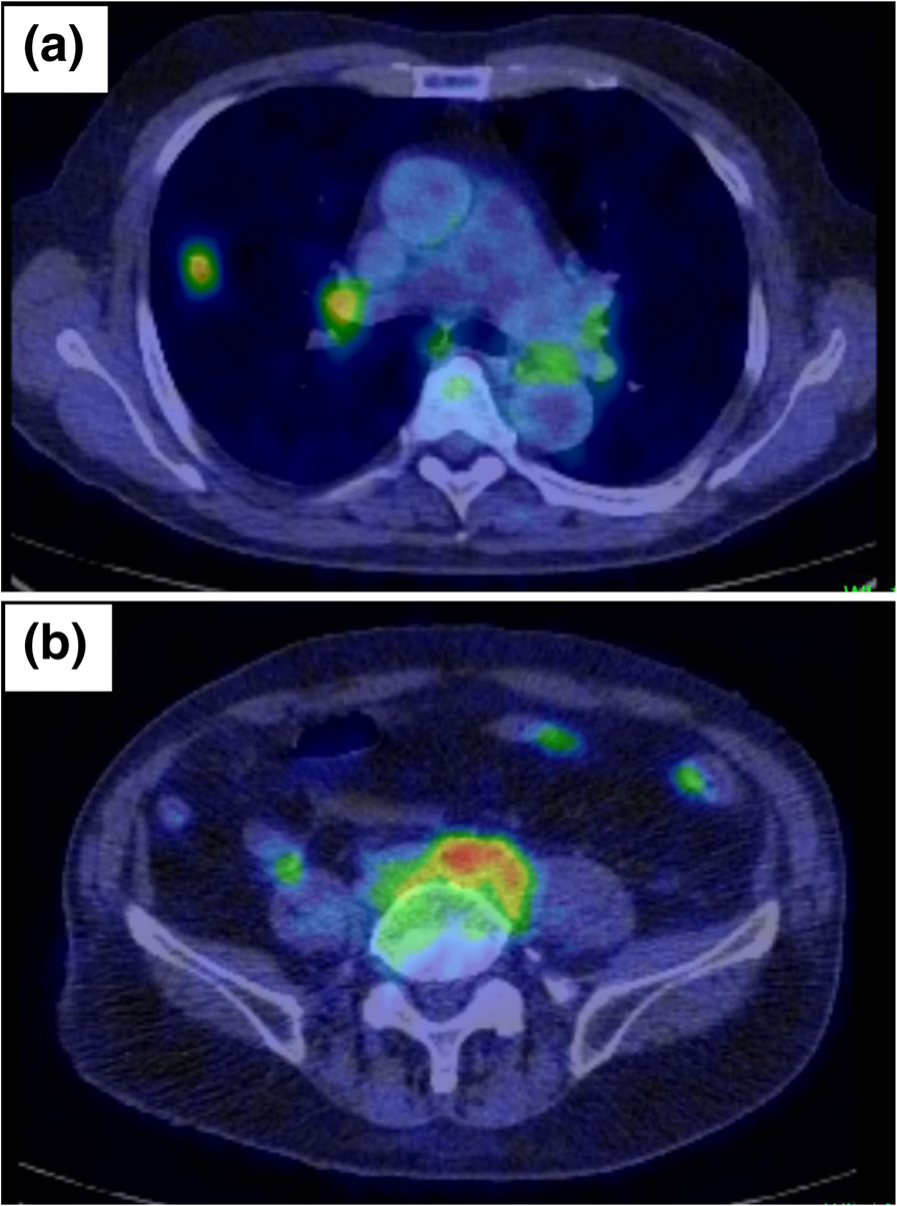
Positron emission tomography-computed tomography examination in a 72-year-old man with coexisting lung cancer and immunoglobulin G4-related disease. There is high accumulation of ^18^F-fluorodeoxyglucose in his **a** lung nodule, hilar, and mediastinal lymph node and in his **b** retroperitoneum

An ^18^F-fluorodeoxyglucose-positron emission tomography (^18^F-FDG-PET) scan revealed high accumulation in his lung nodule, with maximum standardized uptake value (SUV) of 2.7 in the early phase and 3.3 in the delayed phase. His hilar and mediastinal lymph node showed high ^18^F-FDG accumulation with maximum SUV of 2.5 in the early phase and 2.3 in the delayed phase (Fig. 2a). The tumefactive lesion in his anterior sacral spine also showed high ^18^F-FDG accumulation, with maximum SUV of 4.0 in the early phase and 4.5 in the delayed phase (Fig. 2b). Abnormal accumulation in other organs was not observed on ^18^F-FDG-PET. A transbronchial biopsy with bronchoscopy was performed, which revealed carcinoma cells on cytology. He was diagnosed with lung cancer at T1bN0M0, stage 1A; this was suspected to be related to IgG4-related retroperitoneal fibrosis.

**Fig. 3:**
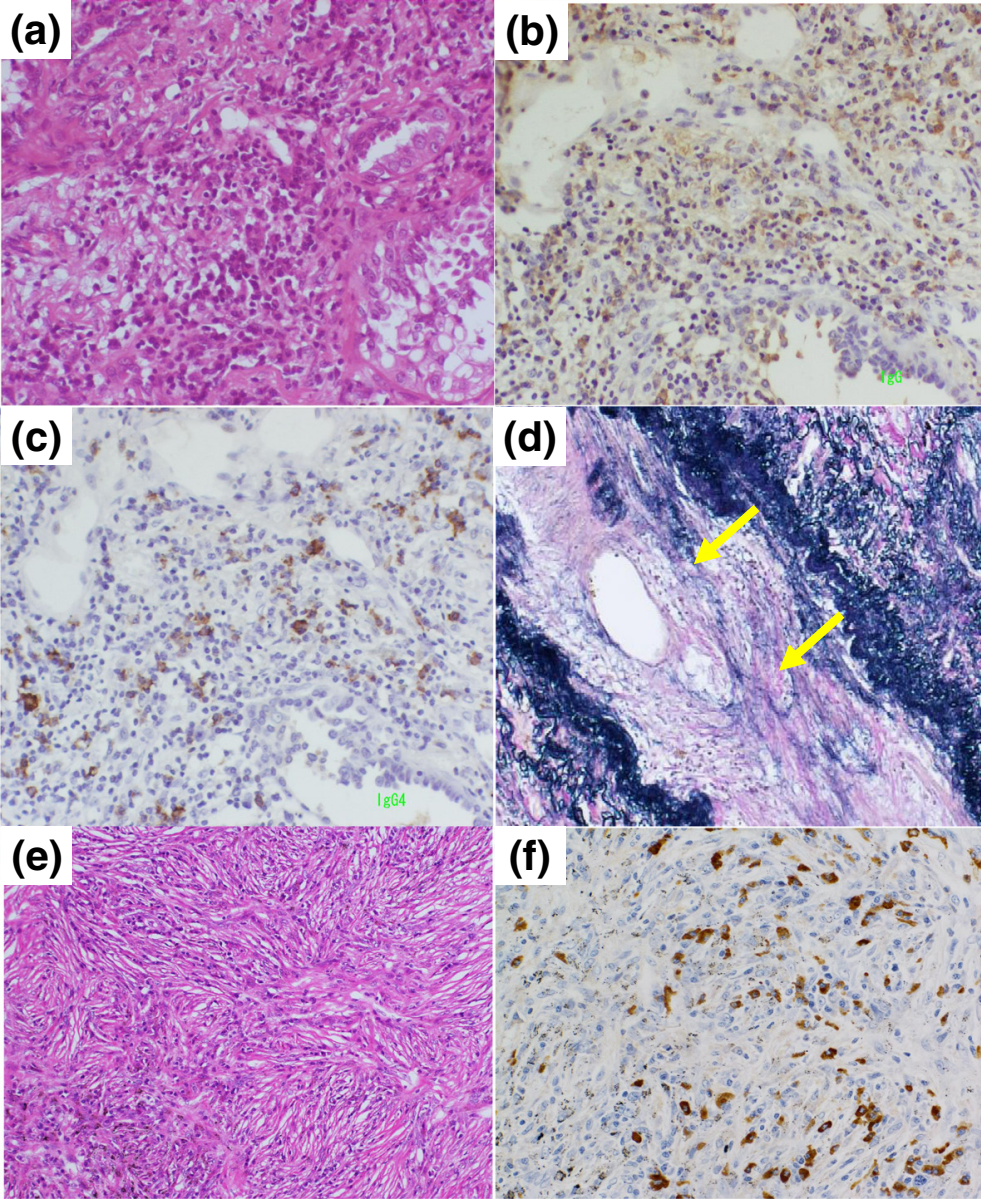
Pathological findings of the resected lung nodule and hilar lymph node in a 72-year-old man with coexisting lung cancer and immunoglobulin G4-related disease. In the lung nodule, **a** lepidic pattern of adenocarcinoma coexisted with lymphocytes and plasma cells (hematoxylin and eosin, ×400). The infiltrating lymphocytes and plasma cells are positive for **b** immunoglobulin G and **c** immunoglobulin G4 staining (×400), with an immunoglobulin G4 to immunoglobulin G ratio of more than 40 %. **d** Obliterative phlebitis findings ("*arrows*") in the lung nodule (Elastica van Gieson, ×200). In the hilar lymph node, **e** the infiltration of plasma cells are seen with storiform fibrosis (×100) and **f** these cells were positive for immunoglobulin G4 staining (×400)

He underwent a right upper lobectomy and regional lymph node dissection. He was discharged on day 10 after lung surgery without any complications. Pathologic findings of the resected lung nodule showed lepidic pattern adenocarcinoma with coexisting infiltration of IgG4-positive plasma cells (Fig. 3a), with an IgG4 to IgG ratio of more than 40 % (Fig. 3b, c). Storiform fibrosis was not seen; however, obliterative phlebitis and nonspecific fibrosis were shown in his lung nodule (Fig. 3d). In his right hilar lymph node which showed positive findings of the ^18^F-FDG-PET scan, infiltration of IgG4-positive plasma cells with storiform fibrosis were seen (Fig. 3e, f). All sampled lymph nodes were negative for cancer cells. Distant metastasis was not observed. At 5 years after operation, he remained free from recurrence of lung cancer.

## Discussion

IgG4-related disease comprises elevated serum IgG4 concentrations and pathologic findings of lymphoplasmacytic infiltration of IgG4-positive plasma cells with storiform fibrosis and obliterative phlebitis in various organs [1]. IgG4-related disease may involve multiple organs and present as autoimmune pancreatitis, Mikulicz’s disease, Riedel’s thyroiditis, retroperitoneal fibrosis, and multifocal fibrosclerosis. Although IgG4 plays a key role in the pathogenesis of the disease, the mechanisms of IgG4 elevation are not understood. It has been speculated that T cells are associated with pathogenesis, because many CD4 T cells are seen at sites of inflammation in IgG4-related disease [2].

Lung involvement of IgG4-related disease often affects the interstitium, airways, mediastinum, and pleura [3]. Common radiologic findings include hilar and mediastinal lymphadenopathies, thickness of bronchovascular bundles, peribronchovascular consolidation, and lung nodules [4]. Fujinaga *et al*. reported that thoracic abnormalities on CT included hilar and mediastinal lymphadenopathies in 78 % of cases; nodular lesions, 3 to 26 mm in size, in 39 % of cases; bronchial wall thickening in 30 % of cases; interlobular thickening in 15 %; and consolidation in 4 % [5].

^18^F-FDG-PET can be used for diagnosis of malignancies because of its capacity to detect metabolic activity in malignant cells. IgG4-related disease has also been reported to demonstrate high ^18^F-FDG uptake in diseased sites [6]. Although the value of SUV may increase in both inflammatory and malignant lesions, PET-CT remains a useful test to identify the extent of a lesion in IgG4-related disease.

The present case showed retroperitoneal fibrosis, lymphadenopathies, and lung nodules with elevated serum IgG4 concentration. Nodular shadow of the lung was also reported to be a major manifestation in 31 to 46 % of IgG4-related lung diseases [5, 7, 8]. In this case, pathologic examination confirmed the coexistence of lung adenocarcinoma and IgG4-positive plasma cells with obliterative phlebitis in a lung nodule. Previous reports suggested that patients with IgG4-related disease had a higher incidence of malignancies, including lung cancer, pancreatic cancer, colon cancer, and malignant lymphoma [9]. Inoue *et al*. reported a similar case of lung cancer and IgG4-related disease [10]. The present case is the second report that cancer cells and IgG4-positive plasma cells coexisted with obliterative phlebitis in the same nodule, rather than being just a complication. It is suggested that the production of IgG4 plasma cells may be a response to unknown antigens. Further research is needed to investigate the pathogenesis of IgG4-related disease.

## Conclusions

We reported a rare case of lung cancer coexisting with IgG4-related disease in the same lung nodule. Pathologic correlation by bronchoscopy and/or surgery should be considered in cases of suspicious IgG4-related disease appearing in a lung nodule.

## Consent

Written informed consent was obtained from the patient for publication of this case report and accompanying images. A copy of the written consent is available for review by the Editor-in-Chief of this journal.

## Abbreviations

CT: computed tomography
^18^F-FDG-PET: ^18^F-fluorodeoxyglucose positron emission tomography
SUV: standardized uptake value
